# Global distribution and climate sensitivity of the tropical montane forest nitrogen cycle

**DOI:** 10.1038/s41467-022-35170-z

**Published:** 2022-11-30

**Authors:** Justin D. Gay, Bryce Currey, E. N. J. Brookshire

**Affiliations:** 1 Department of Land Resources and Environmental Science, Montana State University, Bozeman, MT 59717 USA; 2 Nelson Institute for Environmental Studies, University of Wisconsin – Madison, Madison, WI 53706 USA

**Keywords:** Element cycles, Ecosystem ecology

## Abstract

Tropical forests are pivotal to global climate and biogeochemical cycles, yet the geographic distribution of nutrient limitation to plants and microbes across the biome is unresolved. One long-standing generalization is that tropical montane forests are nitrogen (N)-limited whereas lowland forests tend to be N-rich. However, empirical tests of this hypothesis have yielded equivocal results. Here we evaluate the topographic signature of the ecosystem-level tropical N cycle by examining climatic and geophysical controls of surface soil N content and stable isotopes (δ^15^N) from elevational gradients distributed across tropical mountains globally. We document steep increases in soil N concentration and declining δ^15^N with increasing elevation, consistent with decreased microbial N processing and lower gaseous N losses. Temperature explained much of the change in N, with an apparent temperature sensitivity (*Q*_10_) of ~1.9. Although montane forests make up 11% of forested tropical land area, we estimate they account for >17% of the global tropical forest soil N pool. Our findings support the existence of widespread microbial N limitation across tropical montane forest ecosystems and high sensitivity to climate warming.

## Abstract (web summary)

Tropical montane forests harbor a disproportionately large fraction of the global tropical forest soil N pool. Elevational increases in soil N and decreases in δ15N are largely driven by temperature, suggesting sensitivity of this N pool to warming.

## Introduction

Tropical mountains give rise to some of the most striking climatic and ecological gradients on Earth. Over relatively small distances, elevational gradients in tropical mountains recapitulate latitudinal shifts in temperature^1^ and thus provide a natural laboratory for understanding environmental control of ecosystem function and effects of global change^2,3^. One outstanding question is how climate warming will affect soil nutrient cycles that constrain productivity and carbon storage in tropical montane forests^4–6^. In contrast to most of the lowland tropics, tropical montane forests can accumulate large pools of N in soil organic matter (SOM)^7^, yet are often considered to be limited by the availability of inorganic N^8–10^. Observations and models predict that warming in tropical mountains will enhance primary production^4,11–13^ but also increase losses of soil carbon^3,14^. Increases in microbial N mineralization could further stimulate plant growth but also increase N losses, including emissions of the heat-trapping gas nitrous oxide (N_2_O). However, the degree to which organically-bound N will continue to constrain both processes is uncertain.

Despite decades of research showing slower microbial N processing in montane than lowland tropical forest soils^7,15,16^, it remains unclear how climate variation shapes the balance of N inputs and losses that determine the long-term stability of the soil N pool. The elevational distributions of these fluxes (e.g., biological N fixation, gaseous and hydrologic losses) are poorly quantified for tropical montane forests globally. For example, while soil N_2_O emissions have been shown to decline with increasing elevation in some montane forests^17,18^, consistent with lower N availability, observations of large hydrologic losses of N from other montane forests^19,20^ suggest high N availability at the ecosystem level. Furthermore, the size and geographic distribution of tropical montane forest soil N pools are poorly constrained. Although global inventories show larger soil N pools in tropical mountains than lowlands^21–23^, these estimates are highly uncertain due to limited sampling in forested mountainous terrain^23^. Tropical mountains are distributed across distinct biogeographical regions and a wide range of lithologies and precipitation regimes^24,25^, making generalization difficult. For example, erosion in steep terrain can rejuvenate rock-derived nutrients^26^, including N^27^, but also limit N accumulation, especially in geologically young mountains^28,29^. In addition, regional and elevational variation in precipitation can constrain primary production, SOM decomposition and nutrient availability^2,30,31^. Therefore, disentangling climate versus other biophysical drivers is essential for understanding potential warming effects on the N cycle in tropical montane forests.

Here, we address these uncertainties by analyzing data on soil N and its natural abundance isotope (^15^N) collected across tropical elevational transects globally. We focus our analysis on shallow (0–20 cm) mineral soils as these have shorter SOM residence times and higher climate sensitivity compared to deeper soil^32^ and represent the vast majority of samples collected globally. We use δ^15^N as an integrative measure of ecosystem-level N cycling as the relative abundance of ^15^N to ^14^N in mineral soils records the long-term imprint of microbial processing and N bioavailability^33,34^. In biologically active soils, higher (isotopically enriched) δ^15^N values indicate increased availability of N to plants and microbes and proportionally larger fractionating losses of N (i.e., via gaseous losses)^31,33,35^. While previous global analyses^31,36,37^ show higher soil δ^15^N in warmer and lower precipitation climates, it is unclear how this applies to humid tropical mountains where precipitation shows no globally consistent pattern with elevation^2,25^. Here, we show that temperature and temperature-precipitation interactions control the elevational distribution of the tropical forest soil N cycle. We further show that tropical montane forests harbor a disproportionately large fraction of the global tropical soil N pool.

## Results and discussion

### Elevational patterns of soil N and δ^15^N

**Fig. 1:**
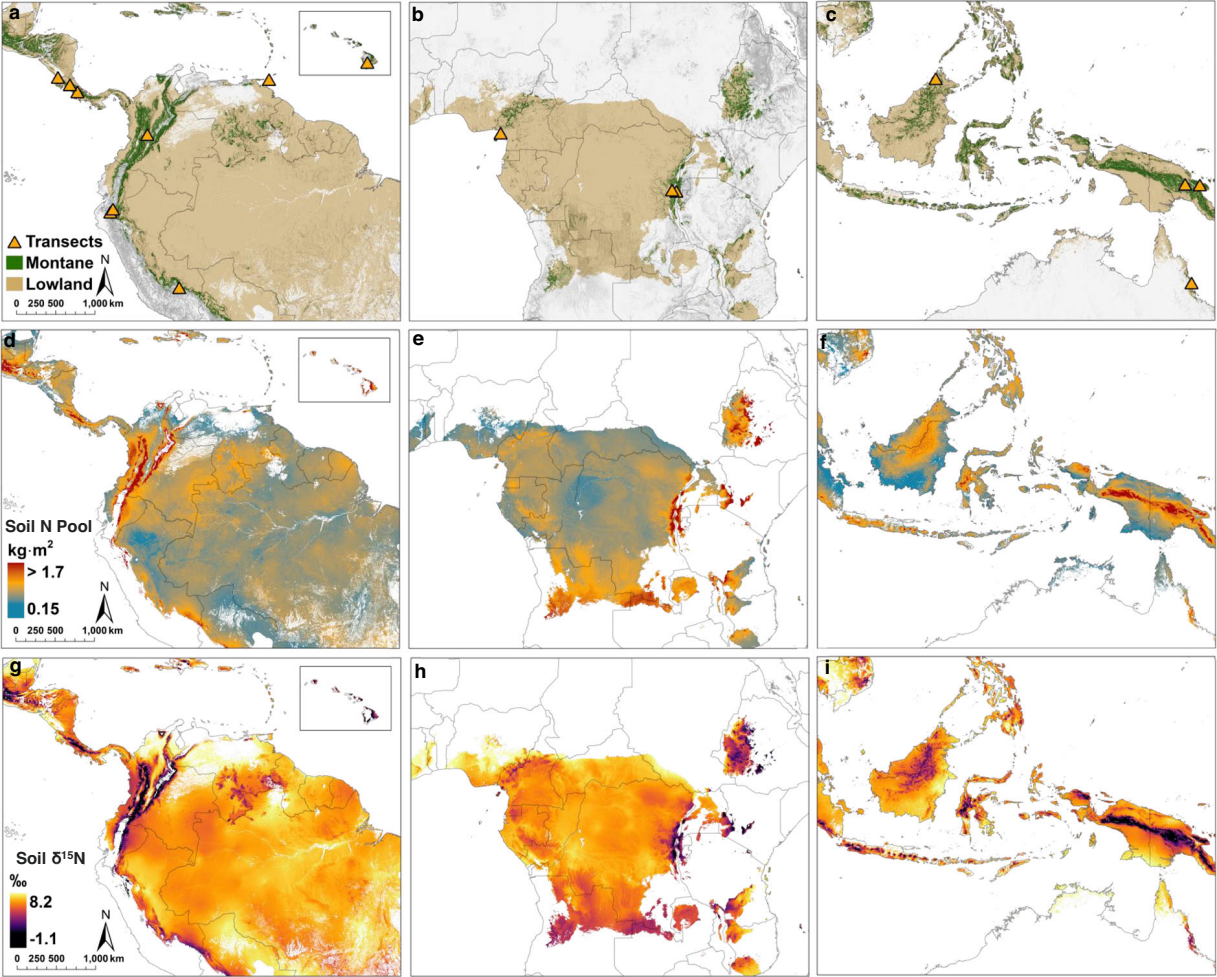
Global distribution of tropical montane forests, soil N pools, and δ^15^N. Extent of montane and lowland tropical forests and locations of elevational gradient transects (**a**–**c**). The distribution of surface mineral soil (0–20 cm) N pools (**d**–**f**) and δ^15^N (**g**–**i**) based on linear mixed-effect models (Supplementary Table 4). Panels display area-equivalent forest extent for regions containing elevational transects. Areas shaded dark gray in the top row represent higher elevations. Areas that are white in second and third rows represent non-forested land areas.

**Fig. 2:**
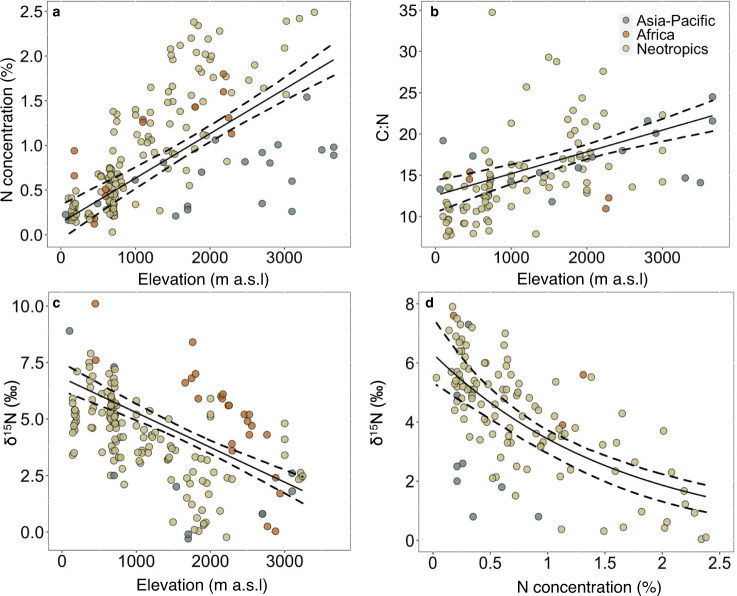
Elevational distribution of soil N concentration, C:N ratios, and δ^15^N across tropical montane forests. The change in soil N concentrations (**a**), C:N (**b**), and δ^15^N (**c**) with increasing elevation were determined using linear mixed-effect models with elevation as the fixed effect and transect identity nested within tropical region as a random effect (Supplementary Table 2). **d** negative exponential relationship between soil δ ^15^N and N concentration. All data presented are from the 16 elevational transects. (**a**) lowland *n* = 33, montane *n* = 141, (**b**) lowland *n* = 26, montane *n* = 86, (**c**) lowland *n* = 27, montane *n* = 141 (**d**) lowland *n* = 22, montane *n* = 100. Model 95% confidence intervals (dashed lines) were estimated using Monte Carlo simulations.

We compiled a database of soil samples from 324 humid forest sites distributed across sixteen elevational gradients (Fig. 1 a-c; Supplementary Fig. 1) and from lowland tropical forests^37^ across the major tropical land areas (Neotropics, Africa, Asia-Pacific). The sites span a large range of elevation [0-3660 meters above sea level (m a.s.l.)], climate [7-30 °C, mean annual temperature (MAT); 1200-8000 mm, mean annual precipitation (MAP)], and a diversity of rock types and lithologic ages (Supplementary Table 1). Environmental conditions across our montane sites (median elevation= 1500 m a.s.l, MAT = 19 °C, MAP = 2460 mm) are broadly representative of global humid tropical montane forests (elevation = 1350 m a.s.l, MAT = 20 °C, MAP = 2050 mm; Supplementary Fig. 2). We first used mixed-effect models to isolate the effect of elevation from any effects of individual transect location (Supplementary Table 2). This revealed large increases in soil N content with increasing elevation across tropical mountains worldwide, although there was considerable regional spread in the data at higher elevations (Fig. 2a). Soil organic carbon (SOC) also increased with elevation (Supplementary Fig. 4), more so than N, resulting in increasing C:N ratios with greater elevation (Fig. 2b). These relationships remained highly significant after removing samples with SOC > 18% (Supplementary Table 3), a threshold generally accepted to indicate the delineation between the organic and mineral layers (FAO World Reference Base for soil classification). Across the global elevational range, soil C:N ratios cross the stoichiometric threshold that governs net N mineralization versus immobilization (C:N ~20-25)^38^, consistent with microbial N limitation and slower bioavailable N cycling in montane compared to lowland forests^5,39^.

Elevational patterns in δ^15^N further support decreasing N bioavailability and microbial processes that discriminate against ^15^N (e.g., nitrification and denitrification) with increasing elevation. Globally, δ^15^N decreases by about 2‰ for every 1 km increase in elevation (Fig. 2c). The range of soil δ^15^N (−0.3 to 10.1‰) and its change with elevation is similar to that documented for soil δ^15^N across global latitudinal climate gradients^36,37^. Additionally, our findings mirror the inverse relationship between the C:N stoichiometry of SOM and its δ^15^N enrichment observed globally^37,40^. Across elevational gradients, δ^15^N declines with increasing soil N concentrations (Fig. 2d), C:N ratios, and N pools (Supplementary Fig. 4). In general, lowland forest soils are characterized by uniformly low total N content and enriched δ^15^N while montane forest soils have high N content and low δ^15^N. This pattern is consistent with a topographic shift from net microbial mineralization to immobilization and lower fractionating loss (e.g., denitrification) as a primary regulator of N accumulation in tropical montane forests.

### Drivers of tropical soil N content and δ^15^N

To determine drivers of N concentrations and δ^15^N, we combined globally gridded datasets of climate, geophysical, and soil properties with transect and lowland data in mixed-effect models. Across all transects, MAT decreases by 5.1 ± 0.07 °C for every 1 km rise in elevation (Supplementary Fig. 5). Hierarchical partitioning analysis (Supplementary Table 4) determined MAT to be the most important driver of soil N concentrations, explaining 41% of the geographic distribution, followed by MAT:MAP interaction (32%), and MAP (27%). For δ^15^N, the model similarly identified MAT as the most important driver (66%), followed by MAP (34%). Both models accurately predict observations with low mean absolute error (Supplementary Fig. 6). These results are consistent with continental and global-scale analyses describing the importance of climate in governing the spatial distribution of the N cycle and its isotopic imprint^33,37,41^, with δ^15^N generally increasing with increasing MAT and decreasing with increasing MAP. MAP is highly variable across tropical regions (Supplementary Fig. 1), but has no coherent relationship with elevation, soil N, or δ^15^N across our sites (Supplementary Figs. 7 and 8). In contrast, the uniformity of the positive MAT-δ^15^N and negative MAT-N relationships across the global diversity of tropical mountain environments points to temperature as a main control of microbial N transformations that isotopically discriminate against δ^15^N.

**Fig. 3:**
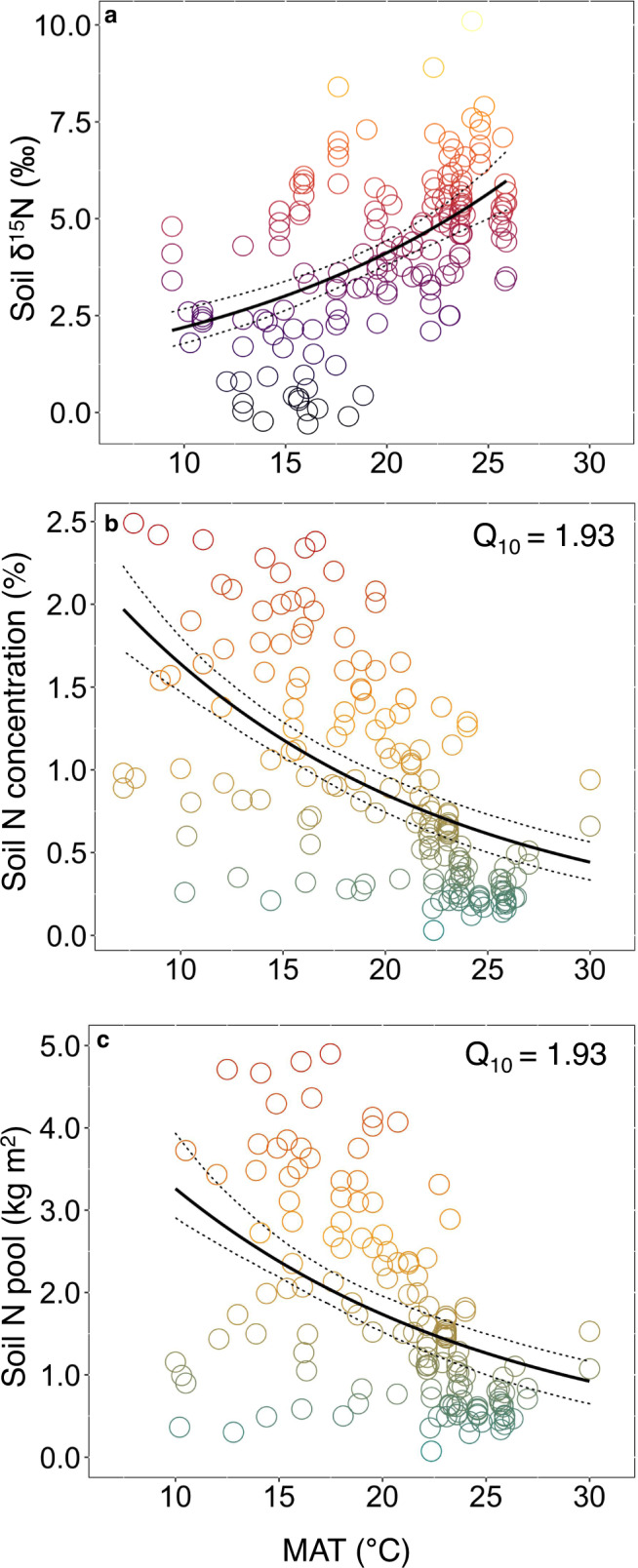
Temperature sensitivity of tropical montane forest N. Curves were fit using nonlinear regression of δ^15^N (**a**), soil N concentrations (**b**) and N pools (**c**) against MAT across all elevational transects. Temperature sensitivities (*Q*_10_) were then determined for (**b**) and (**c**) using regression model results (Methods). Models were fit using elevational transect data and additional lowland sites from a global tropical soil dataset^37^. (**a**) Lowland *n* = 112, montane *n* = 160, (**b**) lowland *n* = 119, montane *n* = 158 (**c**) lowland *n* = 117, montane *n* = 154. The color scale follows Fig. 1.

We next examined the temperature sensitivity of soil N by deriving the apparent *Q*_10_ (i.e., the decrease in N with a 10 °C increase in temperature) using exponential fitting to the elevational variation in MAT (Fig. 3). Consistent with non-linearly coupled changes in N and δ^15^N with elevation, our analysis yielded apparent *Q*_10_ values of ~1.9 for changes in soil N with ambient temperature. This value is consistent with that found for soil enzyme activities (*V*_max_ 1.5-2) measured across tropical elevational gradients^3,42^ and results from litter decomposition^30^ and soil nitrogen mineralization studies^43^ across tropical forests. The global convergence of temperature sensitivities for soil N content and δ^15^N suggests a strong kinetic underpinning of the inverse relationship between soil N accumulation and fractionating loss pathways (i.e., gaseous versus hydrological), consistent with decreasing microbial denitrification with increasing tropical elevation^17,18^. However, such space-for-time analyses cannot determine microbial temperature sensitivity at a given elevation/ambient temperature^14^, nor account for potential acclimation to past or future temperatures^42^. For instance, temperature sensitivities of microbial processes tend to be higher in colder environments^44,45^, suggesting greater temperature sensitivity of microbial N turnover in montane forests than indicated by our gradient analysis.

### Global distribution of tropical montane forest soil N pools and δ^15^N

Based on differences in parameters of elevational changes in N and δ^15^N, we developed geospatial models combining climate, topography (Supplementary Tables 4–7), and tree cover data to map the pantropical distribution of soil N pools and δ^15^N (Fig. 1d-i; Methods). We next calculated soil N pools (0- 20 cm) across our sites by spatially coupling soil N data with globally gridded maps of bulk density (Supplementary Fig. 9). Despite decreasing soil bulk density with increasing elevation, soil N pools are nearly twice as large in montane [1.6 (geometric σ: 2.1) kg N m^−2^] than in lowland forests [0.83 (geometric σː 2.0) kg N m^−2^, two-sided *t* test, t(53.5) = 4.73, *p* = < 0.0001]. Drivers explaining the spatial distribution of soil N pools were similar to that for N concentrations except that MAP emerged as a more important explanatory variable (48%; Supplementary Table 3), followed by MAT (32%), and MAT:MAP interaction (20%). Despite montane forests accounting for only 4.4% (2.1 × 10^6^ km^2^) of tropical land area and 49% of tropical mountainous terrain (Supplementary Tables 5 and 6), we estimate they account for an outsized proportion (17.1%; 1.7 ± 1.3 Pg N) of the global tropical forest soil N pool (10.1 ± 7.7 Pg N; Table 1).

**Table 1:** Total mineral soil N pool for global tropical forests

Region	Total forest soil N (Pg N)	Montane soil N (Pg N)	Lowland soil N (Pg N)
*Neotropics*	4.6 ± 3.5	0.6 ± 0.8	4.0 ± 3.0
*Africa*	3.0 ± 1.8	0.3 ± 0.7	2.8 ± 2.1
*Asia Pacific*	2.4 ± 2.3	0.8 ± 0.6	1.6 ± 1.2
*Global*	10.1 ± 7.7	1.7 ± 1.3	8.4 ± 6.3

N Pool estimates (Pg N ± 95% confidence interval; 0-20 cm) are summed from global models used to create Fig. 1.

Accurate estimates of soil N and its environmental drivers are critical for benchmarking coupled C-N cycles in terrestrial ecosystem models. Although existing global maps also show higher soil N in montane relative to lowland tropical forests, the size and geographic distribution of the montane soil N pool are poorly constrained due to low sampling density^21,23^. For example, soil N concentrations in our montane dataset are 1.6 times higher than simulated by a recent globally gridded data product^23^ for the same sites (Supplementary Fig. 10). Our models also reveal significant geographic variation in the contribution of montane forests to tropical soil N pools (Table 1 and Supplementary Table 6) not captured in existing estimates. For instance, the Asia-Pacific region comprises only a fifth of global tropical land area but holds over half of tropical montane forests and 46% of the global tropical montane forest soil N pool. In contrast, our analysis shows higher soil N concentrations in the Neotropics and higher δ^15^N in Africa (Fig. 2), but it is unclear how lower sample representation in Africa and the Asia-Pacific influence this result. Further, because lowland forests constituted a small proportion of our dataset, our estimates of lowland soil N pools and δ^15^N and their environmental drivers are less certain. This is particularly true for extensive SOC-rich wetland and peatland forests^46^ of western Amazonia, the upper Congo, and southern Borneo (Supplementary Fig. 1). Despite these uncertainties, however, our model estimates of the global tropical soil N pool are similar to the previous estimates^21^ and indicate that montane tropical forests contribute more than proportionally to the global tropical forest N pool.

The prominent role of elevational climate variation in regulating the tropical soil N cycle offers insight into existing conceptual theory. Despite decades of thought that plant growth in montane forests is predominantly limited by N, recent syntheses of tropical fertilization experiments^47^ find no global tendency for trees to be more N limited at higher than lower elevations, and observations of large leaching losses of bioavailable N from some montane forests^19,20^ suggests N richness at the ecosystem scale^48^. Broad geographic evidence for increasing N constraints to microbial but not vegetative activity with elevation suggests some degree of decoupling in the plant-microbe N cycle that may also be subject to change with increased temperature. Although our analysis accounts for broad variation in biophysical drivers, other factors could contribute to the observed patterns in soil N. For example, our analysis is limited to the bulk soil N pool, but the susceptibility of different fractions of the soil N pool (e.g., particulate vs. mineral-associated) to climate change may also depend on elevation. In addition, some tropical montane forests harbor ectomycorrhizal tree species that can potentially influence soil C accumulation, C:N ratios, and N availability^49^. Further, erosional supply of geologic N is higher in mountainous terrain^27^ and could contribute to enhanced N storage. Nevertheless, our isotopic evidence is consistent with increasing microbial N limitation at higher elevations^39^ and a shift in the proportion of N lost from highly fractionating (e.g., denitrification) to less fractionating (e.g., hydrologic leaching) processes^31^.

Our results identify a large and potentially vulnerable pool of soil nitrogen across tropical montane forests. Montane forests are increasingly subject to the direct effects of agricultural expansion and from deforestation-driven amplification of warming^50^. Regional warming is occurring at a faster rate in mountains than lowland tropical land areas^51^, potentially jeopardizing these large stocks of soil organic N via enhanced microbial processing. While our findings reveal emergent climate sensitivities of the soil microbial N cycle, any net ecosystem-level warming effects will depend on the climate sensitives of multiple N sources, sinks and their interaction, including biological N fixation and plant uptake. The degree to which N availability will limit warming-induced enhancement of montane forest productivity, or alternately result in enhanced N losses to water and the atmosphere, will depend on how microbial N turnover and supply keep pace with demand.

## Methods

### Data assembly

We conducted an extensive web-based search for studies reporting mineral soil (<30 cm depth or by horizon) N concentrations (% N) and δ^15^N from mature humid tropical forests (MAP > 1200, latitude ± 23.5° N and S of the equator) where samples were collected along a continuous elevational transect. We first used expert knowledge of tropical elevational transect studies with soil N and/or δ^15^N data and then conducted a Google Scholar search with the terms “tropical montane/mountain forest soil”, “tropical montane/mountain elevational/altitudinal gradient/transect”, “tropical montane/mountain N isotope/15 N/δ^15^N, and “tropical montane/mountain forest nitrogen cycle/cycling”. In total, 16 independent elevational gradient transects were identified for a total of 174 soil N concentration and 168 soil δ^15^N observations. Natural abundance isotope ratios (δ^15^N) are reported as ‰ = [(R_sample_/R_standard_)-1] x 1000, where R = ^15^N:^14^N of the sample and the standard, with the standard as the background atmospheric ratio of ^15^N:^14^N. Five transects included both bulk soil δ^15^N and N concentrations (Costa Rica^19,52^, Trinidad^53^, Borneo^54^, Australia^45^), four transects reported only δ^15^N (the Democratic Republic of the Congo^55^, Rwanda^24^, Ecuador^17,24^), and seven reported only N concentrations (Cameroon^56^, Colombia^7^, Costa Rica^15^, Hawaii^9^, Peru^5^, Papua New Guinea^57,58^; Supplementary Table 1). Two of the sixteen transects were established as part of previous sampling campaigns at Volcan Orosi in Costa Rica^19^ and the Northern Range of Trinidad^53^. Here, we collected and composited four mineral soil samples (0-20 cm) from 33 and 37 locations distributed across elevation. We included an additional 85 lowland sites from a global soil N concentration and δ^15^N dataset^37^ previously aggregated to 0.1° latitude and longitude. While reported sampling depths varied across studies, we excluded all soils >30 cm depth and those classified as organic horizons (Oi, Oa, Oe). However, we cannot rule out the possibility that some studies incorporated organic soils in cases where sampling procedures were not described in detail. Further, it is possible that differences in analytical methods for the determination of N (e.g., Kjeldahl digestion, Dumas combustion) may have influenced our analysis. Geolocation data was requisite for all N concentrations and δ^15^N values to extract gridded ancillary data. For studies^9,15,56,58^ that provided a map figure with no sampling geolocation data, coordinates were derived by georeferencing map figures and extracting the coordinates from the map. For studies without open-source raw data or summary tables, figures were digitized to extract values with *Plot Digitizer* (version 2.6.8.).

### Quantifying tropical montane forest extent

Montane terrain was defined using a six-tier geospatial classification framework^59^ combining slope, elevation, and local elevation range (LER) using a ~1 km cell resolution global DEM (USGS GTOPO30). LER is calculated as the difference between the maximum and minimum elevation within a 5 km radius of each grid cell to identify areas of low elevation but high relief (i.e., rugged topography). Montane land surfaces are pixels with an elevation > 2500 (m a.s.l.), surfaces between 1500-2499 m a.s.l. with slopes > 2°, and surfaces between the elevations of 1000−1499 m a.s.l. with slopes > 5° or an LER of > 300 m. Between the elevations of 300-999 m a.s.l., montane classification is restricted to LER values > 300 m. All terrain <300 m a.s.l. is classified as non-mountain or lowland. To restrict output to forested areas, we created a pantropical forest mask using 2010 global tree cover^60^ and classified pixels with > 10% tree cover and MAP > 1200 mm as humid tropical forest. Each sampling point in our soil chemistry dataset was then mapped onto this forest layer and classified as montane or lowland. All geospatial analyses were conducted using R (v4.0.3, R Core Team 2022)^61^ and ESRI ArcGIS Pro (ESRI 2019).

### Drivers of soil N and δ^15^N

We fit linear mixed-effect regression models using maximum likelihood (*lme4* and *lmerTest* packages in R)^62,63^ to examine relationships between soil C:N, N concentrations, and δ^15^N and elevation across the 16 transects. A transect-specific identifier variable was used as a random effect in all models to account for violations of independence from sampling within individual transects. 95% confidence intervals were calculated using Monte-Carlo simulations for all figures containing transect-derived data in which model error was randomly simulated around mean response values 1000 times using the standard deviation from which the inner 95% was calculated. Response and predictor variables that did not exhibit normal distributions were log-transformed (log_e_) before statistical analyses. All statistical analyses were conducted in R (v4.0.3, R Core Team 2022)^61^. Significance was set a priori to α = 0.05.

Linear mixed-effect regression models were fit to identify the main drivers in the distribution of tropical forest soil δ^15^N and N concentrations by examining biophysical parameters previously shown to influence N cycling processes (elevation, temperature, precipitation, slope angle, percentage clay, and SOC). SOC was used in models of δ^15^N but not N concentrations due to lack of independence between organic C and N in SOM. N pools were calculated by multiplying reported soil N concentrations and modeled bulk density for 0-20 cm (250 m resolution)^22^. Although MAT and elevation exhibit strong collinearity, both were initially included in the full model to determine their relative explanatory power, although model selection subsequently removed elevation from all models. Model refinement was conducted using a selection criterion of Δ AIC > 2 or greater (*dredge* function in R)^64^. We next used hierarchical partitioning analysis to decompose the relative importance of drivers of δ^15^N and N concentrations in the respective regression models with the *Relaimpo* package^65^. Model variance is reported using the ${R}_{({GLMM})}^{2}$ summary statistic and include both the ${R}_{(c)}^{2}$ (variance explained by the entire model) and ${R}_{(m)}^{2}$ (variance explained by the fixed effect; *MuMin* package^64^). We evaluated error between regression predictions and observed soil N concentrations and δ^15^N using mean absolute error (MAE).

To evaluate the temperature sensitivity of the soil N cycle, we calculated an apparent *Q*_10_ by fitting an exponential function of soil N concentrations to the elevational distribution of MAT. Although δ^15^N increases non-linearly with increasing elevational temperature, we did not evaluate *Q*_10_ due to non-proportionality of δ^15^N values. Least-square estimates of the regression parameters for all nonlinear models and significance tests for the exponential term were derived from the *nlstools* package^66^.

1 $${{{{{\rm{soil}}}}}}\,{{{{{\rm{N}}}}}}\,{{{{{\rm{concentration}}}}}}\,(\%)={(3.158)}^{0.066\cdot {{{{{\rm{MAT}}}}}}}$$

2 $${{{{{\rm{soil}}}}}}\,{{{{{\rm{N}}}}}}\,{{{{{\rm{pool}}}}}}\,({{{{{\rm{kg}}}}}}\,{{{{{{\rm{m}}}}}}}^{2})={(6.545)}^{0.066\cdot {{{{{\rm{MAT}}}}}}}$$

and then using results to calculate *Q*_10_:

3 $${({{{{{{\rm{N}}}}}}}_{2}/{{{{{{\rm{N}}}}}}}_{1})}^{\frac{10}{{{{{{{\rm{MAT}}}}}}}_{2}{-{{{{{\rm{MAT}}}}}}}_{1}}}$$

where N_1_ and N_2_ values represent the soil N concentrations from the curve fit with MAT from Eqs. 1 and 2 and MAT_1_ and MAT_2_ are 10 and 20 °C.

### Mapping soil N pools and δ^15^N

To create pantropical maps of soil δ^15^N and N pools (Fig. 1d-i), we multiplied model coefficients from our statistical models with respective gridded variables, including a binary gridded layer of each tropical region at ~1 km resolution. Pantropical soil N pools were then calculated by summing mapped N pool data for the entire tropics, tropical montane, and lowland forests. Uncertainty around our estimates was derived from the multiplication of model SE and the 95% confidence interval critical t-distribution value (${t}^{*}$) which is a function of model degrees of freedom.

4 $${{{{{\rm{Upper}}}}}}/{{{{{\rm{Lower}}}}}}\,{{{{{\rm{CI}}}}}}={{{{{\rm{N}}}}}}\,{{{{{\rm{pool}}}}}}\pm {{{{{{\rm{t}}}}}}}^{\ast }\cdot {{{{{\rm{SE}}}}}}$$

δ^15^N model results were compared to observed values based on the respective modeled values extracted from the sampled geographic coordinates along the transects. The gridded global N pool model was calculated based on observed N concentrations from the transect and lowland forest datasets and modeled bulk density. Finally, we compared our observations of soil N concentrations to those simulated at 250 m resolution by SoilGrids 2.0 (ref. 23) for the same montane coordinates (Supplementary Fig. 10).

## Supplementary information

Supplementary Information

## Data Availability

The data that support the findings of this study are available in the public open access data repository: https://datadryad.org/stash/share/Ywp2F5vNN9SKWiivJaLf94z4mbBebY0yl0ekLhBZmlE.
