# Supplementary material for: Global distribution and climate sensitivity of the tropical montane forest nitrogen cycle
Source: Nat Commun. 2022 Nov 30;13:7364. doi: 10.1038/s41467-022-35170-z (PMC9712492; doi:10.1038/s41467-022-35170-z)
Supplement: Supplementary file 1 — Supplementary Information [file 41467_2022_35170_MOESM1_ESM.pdf]

Supplementary Information for

Global distribution and climate sensitivity of the tropical montane forest N cycle

Gay, J.D, B. Currey, E.N.J Brookshire

Correspondence to: jbrookshire@montana.edu

This pdf file includes:

Supplementary Table 1: Biophysical characteristics of elevational gradients
Supplementary Table 2: Linear mixed model results for elevational transects
Supplementary Table 3: Linear mixed model results for elevational transects (SOC filtered at < 18%)
Supplementary Table 4: Hierarchical partitioning and global soil N concentrations and $\delta^{15}N$ results
Supplementary Table 5: Montane classification criteria
Supplementary Table 6: Global tropical forest extent
Supplementary Table 7: Previously reported tropical forest extent

Supplementary Figure 1: Tropical forest cover extent, SOC, and MAP
Supplementary Figure 2: Comparison of transect and global montane environments
Supplementary Figure 3: SOC across elevational transects
Supplementary Figure 4: Relationship between soil $\delta^{15}N$ and C:N
Supplementary Figure 5: Elevational change in temperature across tropical montane forests
Supplementary Figure 6: Fitted versus observed $\delta^{15}N$ and N concentrations
Supplementary Figure 7: Relationship between MAP and elevation
Supplementary Figure 8: Change in N concentrations and $\delta^{15}N$ with elevational change in MAP
Supplementary Figure 9: Change in soil bulk density with increasing elevation
Supplementary Figure 10: Comparison of observed and SoilGrid model of soil N concentrations

Supplementary References:1-3

Supplementary Table 1. Biophysical properties of elevational gradients. In soil type and bedrock columns, L represents values for the lowest elevation sampling point along the transect, while M represents mid-elevation, and H represents the highest elevation.

	$\delta^{15}N$ (‰)	N (%)	Elevation (m)	MAT (ºC)	MAP (mm)	Soil Type	Bedrock	Cloud Forest Elevation(m)[1]
Australia[45]	2-8.9	0.16-0.31	100 - 1540	22.3-14.4	3939-7625	L: Loam<br>H: Loamy-sand	Metamorphic sedimentary<br>H: granite	1000-3500
Borneo[54]	-0.3- 4.9	0.21-1.9	700 - 3100	23.1-10.2	2300-2300	L: Oxisol<br>H: Histosol/spodosol	Sedimentary/patchy ultrabasic<br>H: Granite (>3000m)	1500-3500
Cameroon[56]	*N/A*	0.43-1.8	180 - 2180	30-18.9	3000-5000	L:Acrudoxic Hapludands<br>M: Vitric Hapludands<br>H: Thaptic Hulvudands	Recent basaltic lava flows overlying Holocene olivine basalt	1000-3500
Colombia[7]	*N/A*	0.09-1.1	16 - 2994	29.9-11.9	1200-8000	*Not Reported*	*Not Reported*	1800-3500
Costa Rica[19]	2.1-7	0.03-2.08	634 - 1479	23-19.5	1987-3139	Ultisols/Inceptisols	Pleistocene to Holocene basalts	1800-3500
Costa Rica[52]	-0.23-6.1	0.27-2.38	537 - 2289	23-13.9	2438-3648	Ultisols/Humults	Miocene-era igneous	1800-3500
Costa Rica[15]	N/A	0.41-1.96	100 - 2600	24-10.5	2260-5096	L: Entisol<br>H: Andisols	Plio-Pleistocene basalt and andesite	1800-3500
DRC[55]	3.9-10.1	N/A	448-2289	24.2-20	1800-2000	L: Ferralsols<br>H: Ferralsols/Acrisols	Basalt	2000-3500
Ecuador[17]	2.3-5.8	N/A	394 - 3241	23.7-10.9	1241-3720	L: Inceptisols<br>H: Planosols/Histosols	M: Jurassic Granite<br>L: and H: Paleozoic metamorphic shist/sandstone	>400
Ecuador[24]	3.4-5.2	N/A	1001 - 3000	19.4-9.4	1950-4500	Andisols	Lahar/red bed volcaniclastics/granitic	>400
Hawaii[57]	*N/A*	1.25-2.12	750 -1600	18-12	3000-6350	Histosols	3100 y/o basalt flow	1000-3000
Papua-New Guinea[58]	*N/A*	0.89-1.54	3330 - 3660	9.0-7.2	3450	Inceptisol	Pliocene Granodiorite	2000-3500
Papua-New Guinea[59]	*N/A*	0.22-1.06	62-2912	26.3-10	2598-4218	L: Hapludolls/Rendolls<br>M: Troporthents<br>H: Cryorthents	L: Limestone over alluvial deposits<br>H: Limestone	2000-3500
Peru[5]	N/A	0.23-2.49	194 - 3400	26.4-7.7	1633-3967	L:Haplic Alisols/Cambisols<br>M: Cambisols<br>H: Umbrisols	Paleozoic meta sedimentary mudstone<br>M: Granite intrusions	1800-3500
Rwanda[22]	0.03-8.4	N/A	1659-2937	17.6-12.9	1518-1835	Inceptisols/ultisol/entisol	Shale and quartz	2000-3500
Trinidad[53]	3.4-7.9	0.14-0.54	142-727	25.8-23.5	1638-2238	Ultisols	Meta-sedimentary phyllites from Cretaceous and Jurassic and Mesozoic metamorphic	>660

Supplementary Table 2. Linear mixed model results (Figure 2a, 2b, 2c) for N cycling indices (fixed effects) across the elevational transects. Transect ID is nested within tropical region as the random effect.

Response	Effect	Coeff.	SE	P-value	$R^2_{(m)}$	$R^2_{(c)}$	n
N conc (%)	Elevation (m a.s.l)	0.0005	0.00004	<0.00001	0.30	0.78	174
$\delta^{15}N$ (‰)	Elevation (m a.s.l)	-0.0015	0.0002	<0.00001	0.30	0.70	168
C:N	Elevation (m a.s.l)	0.00017	0.00004	<0.00001	0.19	0.62	112
N pool (kg m^2)	Elevation (m a.s.l)	0.0009	0.00009	<0.00001	0.24	0.73	170

Supplementary Table 3. Linear mixed model results for elevational transects (SOC filtered at < 18%). Values filtered at FAO World Reference Base for mineral soil classification. Transect ID is nested within tropical region as the random effect.

Response	Effect	Coeff.	SE	P-value	$R^2_{(m)}$	$R^2_{(c)}$	*n*
N conc (%)	Elevation (m a.s.l)	0.0005	0.000072	<0.00001	0.27	0.74	88
$\delta^{15}N$ (‰)	Elevation (m a.s.l)	-0.0019	0.00029	<0.00001	0.23	0.73	63
C:N	Elevation (m a.s.l)	0.00011	0.000038	0.0047	0.11	0.23	74
N pool (kg m^2)	Elevation (m a.s.l)	0.0005	0.0001	<0.00001	0.21	0.58	87

Supplementary Table 4. Hierarchical partitioning results for drivers of tropical soil N concentrations (%), N pools (kg m^2) and δ^{15}N (‰). Fixed effect coefficients, estimated marginal and conditional R^2, and relative importance metric (Lmg) for drivers [MAT (°C), MAP (mm)] of global δ^{15}N and N pool distributions across tropical forest soils. Lmg metric is normalized to sum to 100%.

Response	Effect	Coeffs.	SE	p-value	$R^2_{(m)}$	$R^2_{(c)}$	Lmg (%)
δ^{15}N	MAT	1.64	0.03	0.001	0.38	0.81	66
	(log)MAP	1.94	0.37	0.13			34
N conc	MAT	-0.95	0.23	<0.00001	0.35	0.71	41
	(log)MAP	-1.94	0.64	0.003			27
	MAT*MAP	0.11	0.03	0.0002			32
N pool	MAT	-0.85	0.23	0.0002	0.25	0.68	32
	(log)MAP	-1.7	0.63	0.006			48
	MAT*(log)MAP	0.10	0.03	0.0008			20

Supplementary Table 5. Montane classification criteria and their binned tropical land area estimates. Numeric values in Description column are elevation in (m a.s.l) and LER is the local elevational range.

**Categories**	**Description**	**Raster Cells**	**Area Coverage (km^2)**	**Proportion of Montane Tropics (%)**
Level 1	< 1000 and LER > 300	1,321,090	1,098,647	25.80
Level 2	1000-1500 and slope > 5º slope angle *or* LER > 300 m	1,152,823	958,712	22.50
Level 3	1500-2500 and slope > 2º	1,429,402	1,188,721	27.90
Level 4	2500-3500	517,098	430,030	10.10
Level 5	3500-4500	559,921	465,642	10.94
Level 6	> 4500	137,249	114,139	2.68

Supplementary Table 6. Global and regional extent of tropical montane forest. All areas in km^2.

Region	Total Area	Forest Area	Montane Area	Montane Forest Area
Neotropics	15,083,978	9,556,500	1,989,714	684,021
Africa	24,654,851	6,067,746	1,204,124	364,898
Asia Pacific	9,717,223	4,440,035	1,218,625	1,101,545
Global	49,456,052	20,064,281	4,412,463	2,150,464

Supplementary Table 7. Previously reported tropical forest extent.

Citation	Montane forest extent	Total tropical forest (M km^2)
Pan et al. 2013[2]	3.51 million km^2	13.5
Wright 2019	12% (tropical mountains)	11
Kapos et al. 2000	~2 million km^2	
FAO 2020[3]	1.79 million km^2	19.9
Bruijnzeel et al. 2011[1]	2.21 million km^2	15.7
This study	2.15 million km^2	20.1

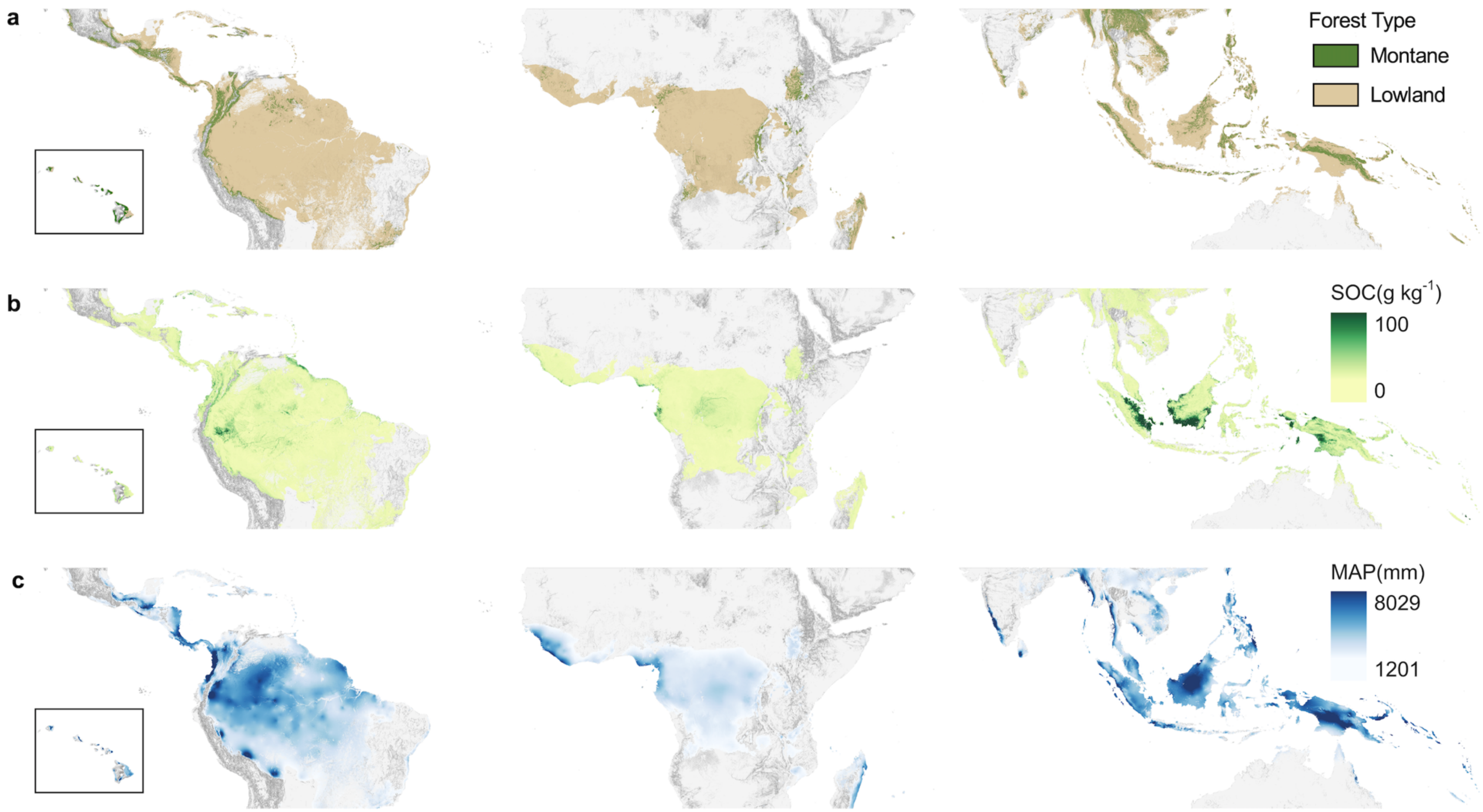

Supplementary Figure 1. Tropical forest cover extent, SOC, and MAP. Grey areas are classified as non-forested ecosystems. Hawai'i insets not to scale.

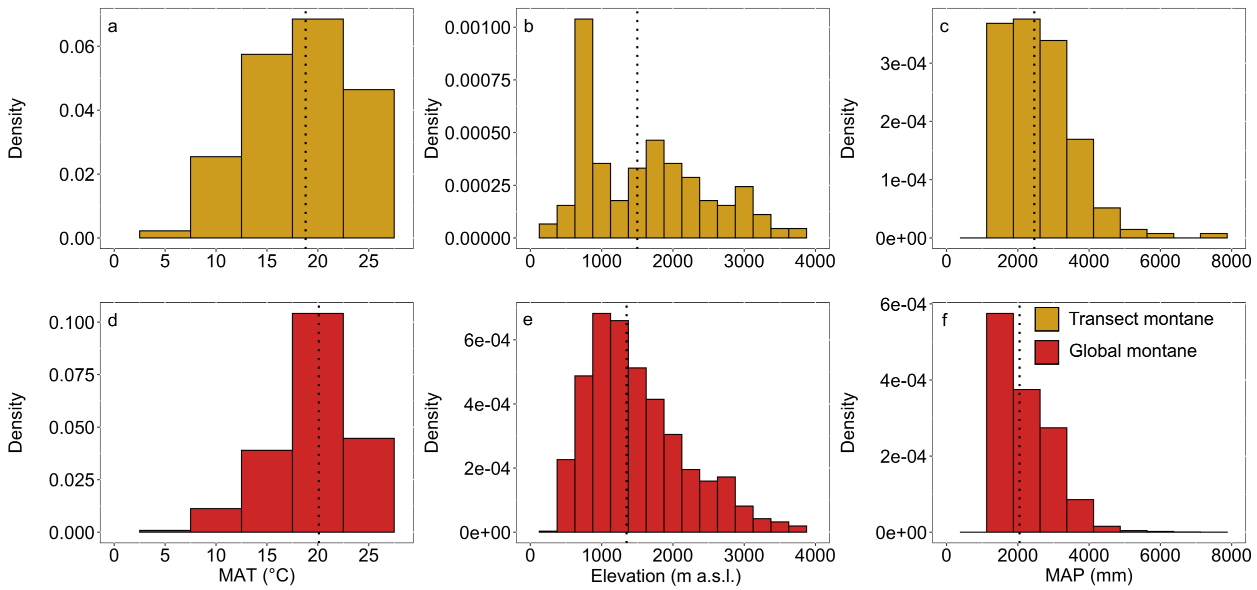

Supplementary Fig. 2. Comparison of elevational transect and global montane environments. Shown are histograms comparing environmental conditions of tropical montane forest elevational gradient sites and global tropical montane forests (n = 1,270,274 pixels). Dotted line represents the median value in each frame.

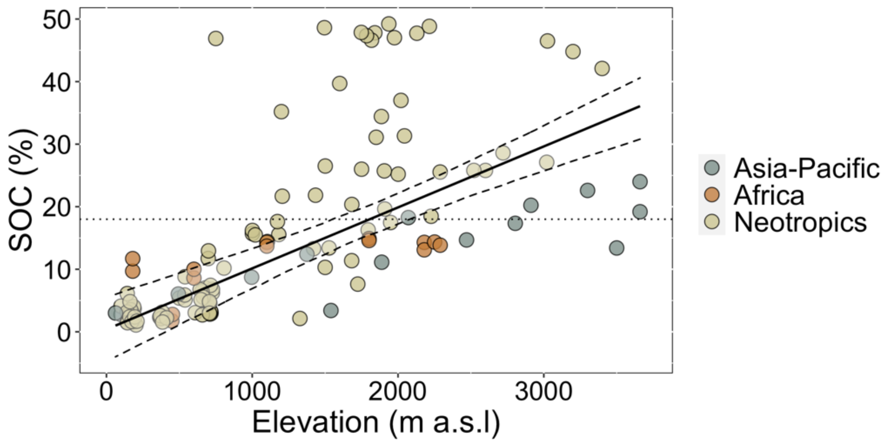

Supplementary Fig. 3. Relationship between soil organic carbon (%) and elevation across the transects. Trend line represents the global mean where SOC increases with elevation (p = <0.00001, R^2m = 0.30, R^2C = 0.74; ±95% CI). Color indicates tropical region. The slope is the calculated fixed effect from a linear mixed model with transect location nested within region. Dotted horizontal line indicates the FAO World Reference base classification criteria for organic soils (SOC > 18%, n = 38), showing that many of the mineral samples from the neotropics had high soil organic matter contents. All data are from the 16 elevational gradients (lowland n = 33, montane n = 93).

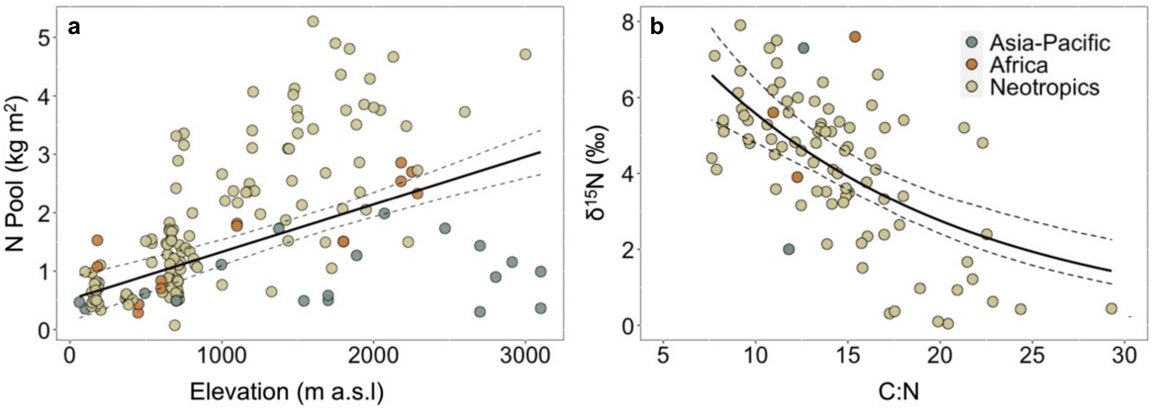

Supplementary Fig. 4. Nonlinear relationship between bulk soil δ^{15}N and soil C:N. The slope in (a) is the calculated fixed effect from a linear mixed model with transect location nested within region as the random effect and dashed lines are 95% confidence intervals that were estimated using Monte-Carlo simulations (Supplemental Table 2). (b) Trend line was fit with a negative exponential curve (δ^{15}N = 10.803 * exp $^{(-0.065* x)}$, $p < 0.00001$) with 95% confidence intervals derived from Monte-Carlo simulations. All data are from the 16 elevational gradients. (a) lowland n = 33, montane n = 137, (b) lowland n = 22, montane n = 68.

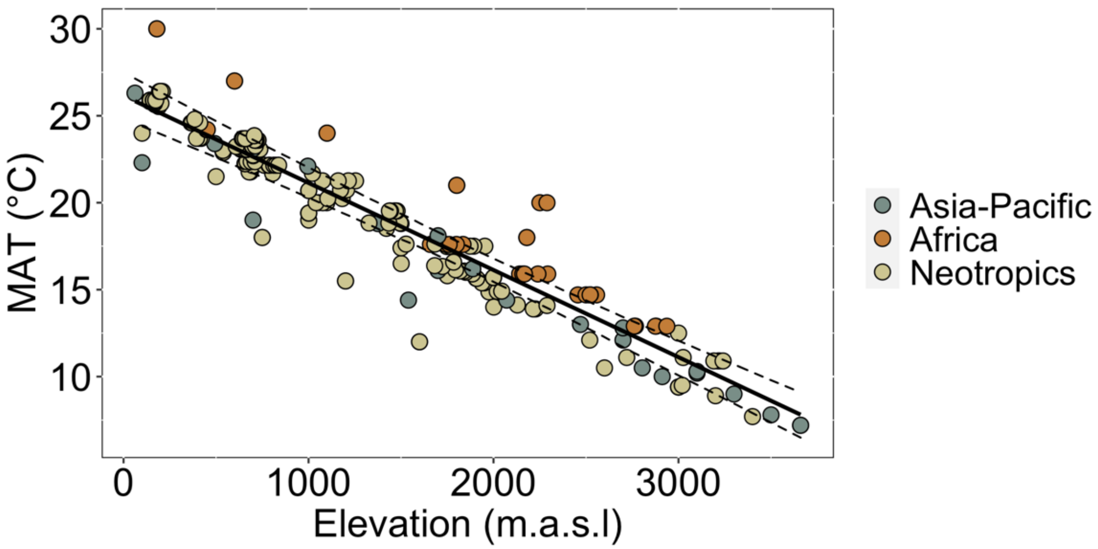

Supplementary Fig. 5. Elevational change in temperature across tropical montane forests. Trend line displays a significant relationship (*p* = <0.00001, R^2_m = 0.81, R^2_c = 0.98 ; ±95% CI) between mean annual temperature and elevation across the transects (5.1 °C km^{-1} lapse rate). The slope is the calculated fixed effect from a linear mixed model with transect location nested within region as the random effect (lowland *n* = 38, montane *n* = 182).

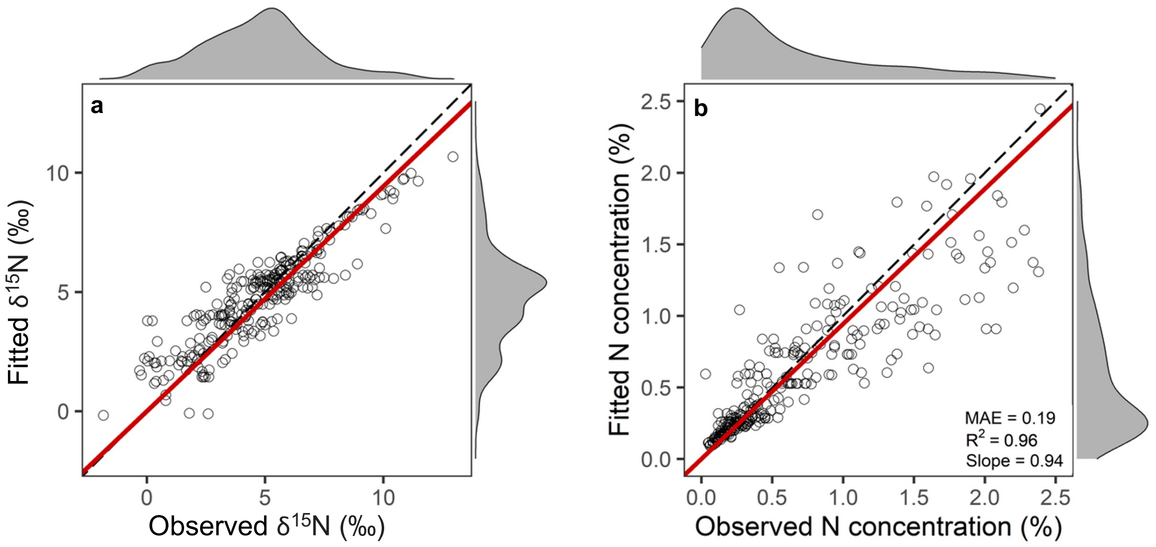

Supplementary Fig. 6. Fitted vs. observed data comparison for a) $\delta^{15}N$ (‰) and b) N concentrations (%). The black dashed line depicts a 1:1 line, and the red line is the slope of the fitted vs. observed data. Fitted data are results from global linear mixed-effects models for the original sampling locations.

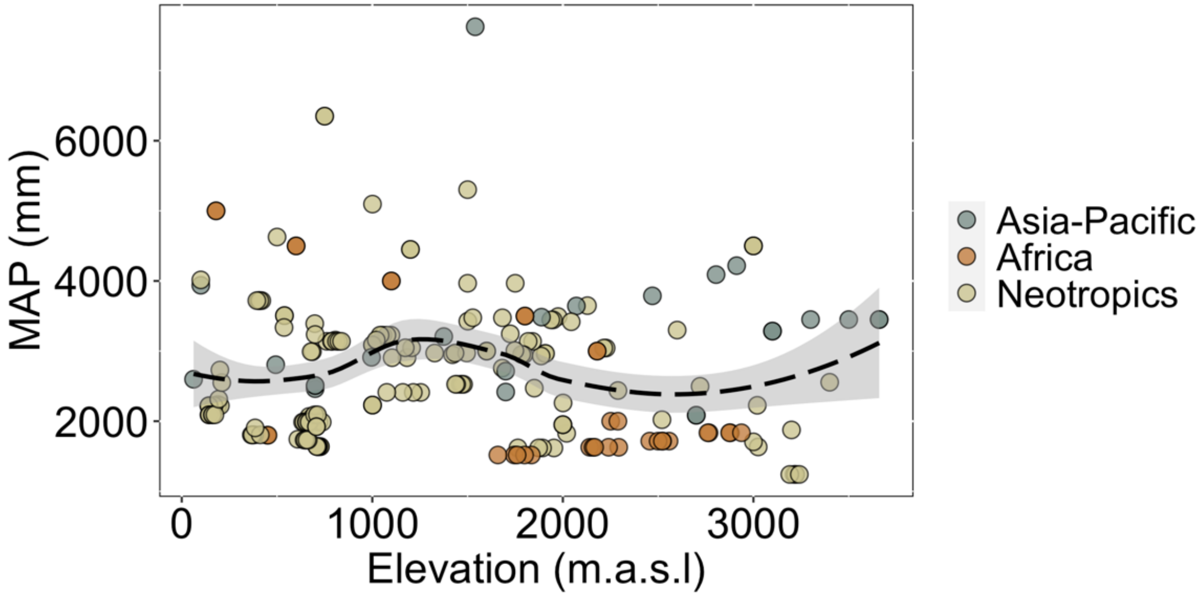

Supplementary Fig. 7. Relationship between mean annual precipitation (mm) and elevation across tropical montane forests. Data were fit with a smoothed LOESS line with shaded area representing the ±95% CI. All data are from the 16 elevational transects (lowland n = 38, montane n = 182).

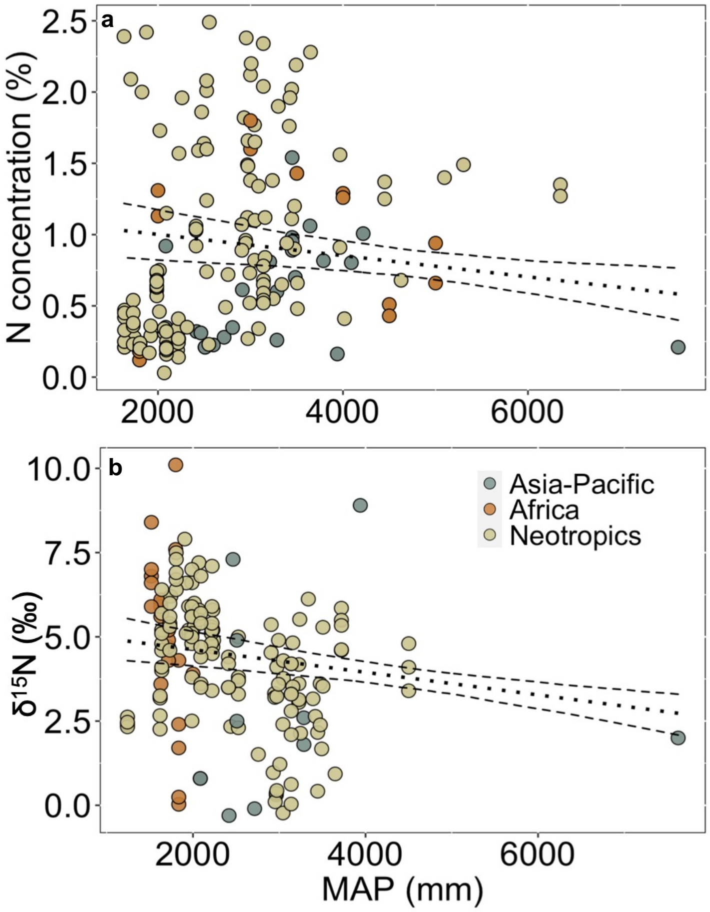

Supplementary Fig. 8. Relationships between soil $\delta^{15}N$ (a), N (b), and mean annual precipitation (mm) across the elevational transects. Dotted trend lines denote non-significant linear relationships $p > 0.05$ with ±95% CI. The slope is the calculated fixed effect from a linear mixed model with transect location nested within region as the random effect. All data are from the 16 elevational gradients (a) lowland $n = 33$, montane $n = 141$, (b) lowland $n = 27$, montane $n = 141$.

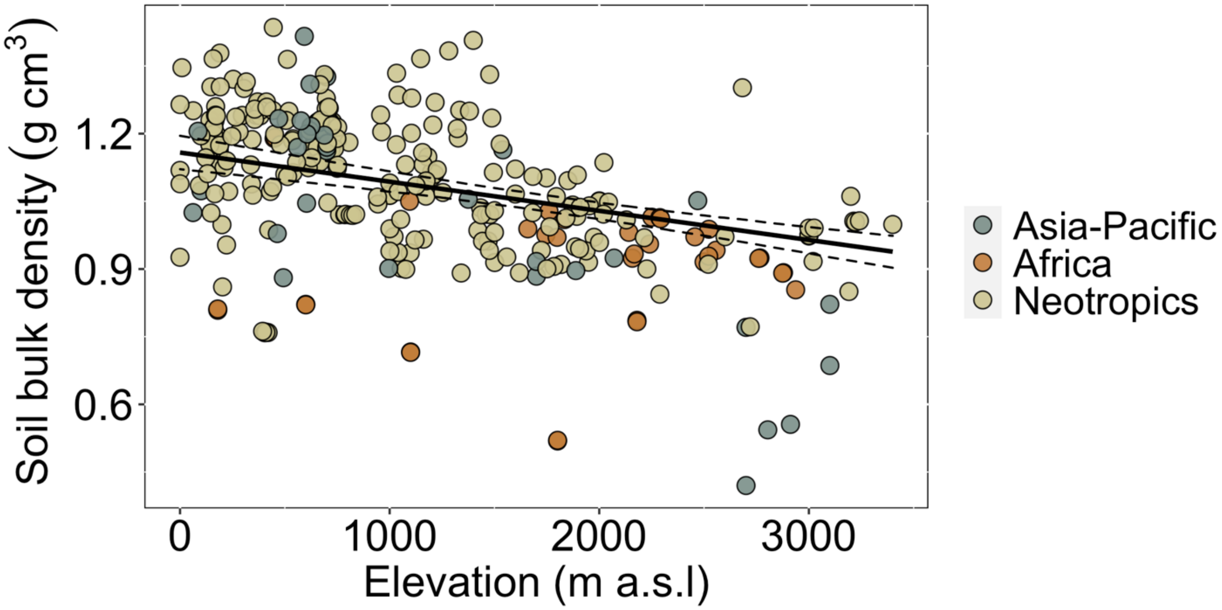

Supplementary Fig. 9. Changes in soil bulk density (g cm^3) across the elevational transects. Trend line displays the global mean linear relationship (p = < 0.00001, R^2m = 0.13, R^2c = 0.64; ±95% CI) between decreasing bulk density (to 20 cm soil depth) and increasing elevation 95%. The slope is the calculated fixed effect from a linear mixed model with transect location nested within region. Data are sourced from transect data and additional lowland sites (n = 318).

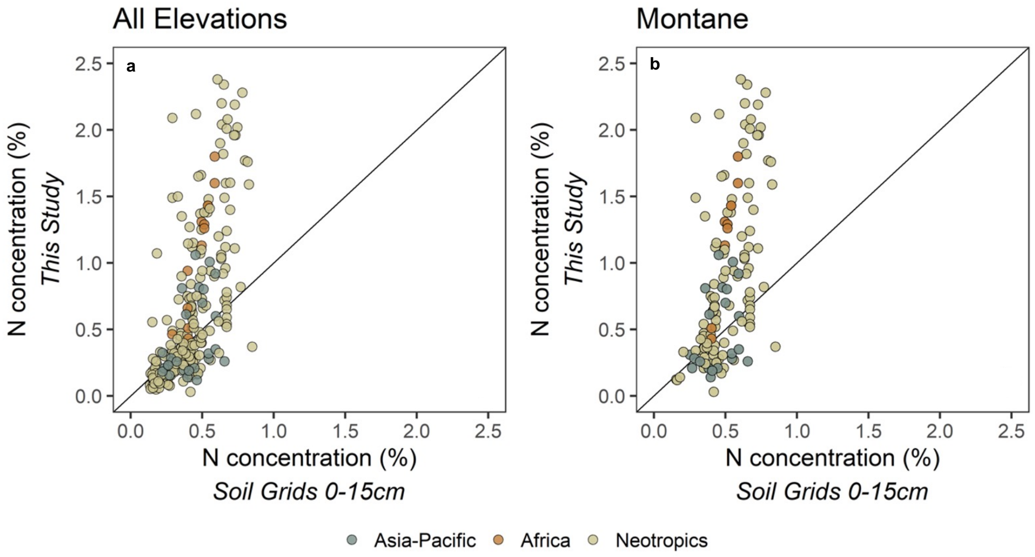

Supplementary Fig. 10. Observed versus SoilGrid 2.0 soil N concentrations (%). Each point shows the observed and simulated (SoilGrids 2.0) soil N concentration for all tropical soil (a) and (b) montane elevational gradient site coordinates. Data are sourced from transect data and additional lowland sites (*n* = 277).

References

1. Bruijnzeel, L. A., Scatena, F. N., & Hamilton, L. S. (2011). *Tropical Montane Cloud Forests Science for conservation and management*. Cambridge University Press.
2. Pan, Y., Birdsey, R. A., Phillips, O. L. & Jackson, R. B. The Structure, Distribution, and Biomass of the World's Forests. *Annual Review of Ecology, Evolution, and Systematics* **44**, 593–622 (2013).
3. FAO. 2020. Global Forest Resources Assessment 2020: Main report. Rome. https://doi.org/10.4060/ca9825en
